# Supplementary material for: Expression of c-MET in Estrogen Receptor Positive and HER2 Negative Resected Breast Cancer Correlated with a Poor Prognosis
Source: J Clin Med. 2022 Nov 26;11(23):6987. doi: 10.3390/jcm11236987 (PMC9738605; doi:10.3390/jcm11236987)
Supplement: Supplementary file 1 [file jcm-11-06987-s001.zip › jcm-2016043-supplementary.pdf]

Table S1. List of pathways and gene ontology terms enriched by c-Met [ENSG00000105976] along with Estrogen Receptor 1 [ENSG00000091831], Estrogen Receptor 2 [ENSG00000140009], Progesterone Receptor [ENSG00000082175], Erb-B2 Receptor Tyrosine kinase 2 [ENSG00000141736]

GO/Pathway	Pathway / Gene Ontology Description	GO/Pathway ID	Adjusted p-value	Gene List
Gene Ontology: Molecular Function	signaling receptor activity	GO:0038023	0,0004	ENSG00000105976,ENSG00000141736,ENSG00000082175,ENSG00000140009,ENSG00000091831
Gene Ontology: Molecular Function	molecular transducer activity	GO:0060089	0,0004	ENSG00000105976,ENSG00000141736,ENSG00000082175,ENSG00000140009,ENSG00000091831
Gene Ontology: Molecular Function	enzyme binding	GO:0019899	0,0023	ENSG00000105976,ENSG00000141736,ENSG00000082175,ENSG00000140009,ENSG00000091831
Gene Ontology: Biological Process	positive regulation of DNA-templated transcription	GO:0045893	0,0028	ENSG00000105976,ENSG00000141736,ENSG00000082175,ENSG00000140009,ENSG00000091831
Gene Ontology: Biological Process	positive regulation of nucleic acid-templated transcription	GO:1903508	0,0028	ENSG00000105976,ENSG00000141736,ENSG00000082175,ENSG00000140009,ENSG00000091831
Gene Ontology: Biological Process	positive regulation of RNA biosynthetic process	GO:1902680	0,0028	ENSG00000105976,ENSG00000141736,ENSG00000082175,ENSG00000140009,ENSG00000091831
Gene Ontology: Biological Process	positive regulation of RNA metabolic process	GO:0051254	0,0041	ENSG00000105976,ENSG00000141736,ENSG000000821

				75,ENSG00000140009,ENSG00000091831
Gene Ontology: Biological Process	positive regulation of macromolecule biosynthetic process	GO:0010557	0,0051	ENSG00000105976,ENSG00000141736,ENSG00000082175,ENSG00000140009,ENSG00000091831
Gene Ontology: Biological Process	positive regulation of cellular biosynthetic process	GO:0031328	0,0067	ENSG00000105976,ENSG00000141736,ENSG00000082175,ENSG00000140009,ENSG00000091831
Gene Ontology: Biological Process	positive regulation of nucleobase-containing compound metabolic process	GO:0045935	0,0069	ENSG00000105976,ENSG00000141736,ENSG00000082175,ENSG00000140009,ENSG00000091831
Gene Ontology: Biological Process	positive regulation of biosynthetic process	GO:0009891	0,0073	ENSG00000105976,ENSG00000141736,ENSG00000082175,ENSG00000140009,ENSG00000091831
Reactome Pathway	Generic Transcription Pathway	REAC:R-HSA-212436	0,0040	ENSG00000105976,ENSG00000141736,ENSG00000082175,ENSG00000140009,ENSG00000091831
Reactome Pathway	RNA Polymerase II Transcription	REAC:R-HSA-73857	0,0064	ENSG00000105976,ENSG00000141736,ENSG00000082175,ENSG00000140009,ENSG00000091831
Reactome Pathway	Gene expression (Transcription)	REAC:R-HSA-74160	0,0103	ENSG00000105976,ENSG00000141736,ENSG00000082175,ENSG00000140009,ENSG00000091831
Gene Ontology: Biological Process	tube morphogenesis	GO:0035239	0,0109	ENSG00000105976,ENSG00000141736,ENSG000000821

				75,ENSG00000091831
Gene Ontology: Biological Process	tube development	GO:0035295	0,0261	ENSG00000105976,ENSG00000141736,ENSG00000082175,ENSG00000091831
Gene Ontology: Biological Process	positive regulation of transcription by RNA polymerase II	GO:0045944	0,0461	ENSG00000105976,ENSG00000082175,ENSG00000140009,ENSG00000091831
KEGG Pathway	Breast cancer	KEGG:05224	0,0000	ENSG00000141736,ENSG00000082175,ENSG00000140009,ENSG00000091831
KEGG Pathway	Pathways in cancer	KEGG:05200	0,0041	ENSG00000105976,ENSG00000141736,ENSG00000140009,ENSG00000091831
Reactome Pathway	Constitutive Signaling by Aberrant PI3K in Cancer	REAC:R-HSA-2219530	0,0000	ENSG00000105976,ENSG00000141736,ENSG00000140009,ENSG00000091831
Reactome Pathway	PI5P, PP2A and IER3 Regulate PI3K/AKT Signaling	REAC:R-HSA-6811558	0,0000	ENSG00000105976,ENSG00000141736,ENSG00000140009,ENSG00000091831
Reactome Pathway	PI3K/AKT Signaling in Cancer	REAC:R-HSA-2219528	0,0000	ENSG00000105976,ENSG00000141736,ENSG00000140009,ENSG00000091831
Reactome Pathway	Negative regulation of the PI3K/AKT network	REAC:R-HSA-199418	0,0000	ENSG00000105976,ENSG00000141736,ENSG00000140009,ENSG00000091831
Reactome Pathway	PIP3 activates AKT signaling	REAC:R-HSA-1257604	0,0003	ENSG00000105976,ENSG00000141736,ENSG00000140009,ENSG00000091831
Reactome Pathway	Intracellular signaling by second messengers	REAC:R-HSA-9006925	0,0005	ENSG00000105976,ENSG00000141736,ENSG00000140009,ENSG00000091831
Reactome Pathway	Diseases of signal transduction by growth factor	REAC:R-HSA-	0,0022	ENSG00000105976,ENSG00

	receptors and second messengers	5663202		000141736,ENSG00000140009,ENSG00000091831
Reactome Pathway	Signaling by Receptor Tyrosine Kinases	REAC:R-HSA-9006934	0,0047	ENSG00000105976,ENSG00000141736,ENSG00000082175,ENSG00000091831
WikiPathway	Mammary gland development pathway - Pregnancy and lactation (Stage 3 of 4)	WP:WP2817	0,0000	ENSG00000141736,ENSG00000082175,ENSG00000140009,ENSG00000091831
WikiPathway	Breast cancer pathway	WP:WP4262	0,0001	ENSG00000141736,ENSG00000082175,ENSG00000140009,ENSG00000091831
Gene Ontology: Molecular Function	nuclear steroid receptor activity	GO:0003707	0,0000	ENSG00000082175,ENSG00000140009,ENSG00000091831
Gene Ontology: Molecular Function	nuclear receptor activity	GO:0004879	0,0000	ENSG00000082175,ENSG00000140009,ENSG00000091831
Gene Ontology: Molecular Function	ligand-activated transcription factor activity	GO:0098531	0,0000	ENSG00000082175,ENSG00000140009,ENSG00000091831
Gene Ontology: Molecular Function	steroid binding	GO:0005496	0,0002	ENSG00000082175,ENSG00000140009,ENSG00000091831
Gene Ontology: Biological Process	intracellular steroid hormone receptor signaling pathway	GO:0030518	0,0013	ENSG00000082175,ENSG00000140009,ENSG00000091831
Gene Ontology: Biological Process	steroid hormone mediated signaling pathway	GO:0043401	0,0022	ENSG00000082175,ENSG00000140009,ENSG00000091831
Gene Ontology: Biological Process	branching morphogenesis of an epithelial tube	GO:0048754	0,0032	ENSG00000105976,ENSG00000082175,ENSG00000091831

Gene Ontology: Biological Process	morphogenesis of a branching epithelium	GO:0061138	0,0055	ENSG00000105976,ENSG00000082175,ENSG00000091831
Gene Ontology: Biological Process	hormone-mediated signaling pathway	GO:0009755	0,0064	ENSG00000082175,ENSG00000140009,ENSG00000091831
Gene Ontology: Biological Process	morphogenesis of a branching structure	GO:0001763	0,0068	ENSG00000105976,ENSG00000082175,ENSG00000091831
Gene Ontology: Biological Process	cellular response to steroid hormone stimulus	GO:0071383	0,0080	ENSG00000082175,ENSG00000140009,ENSG00000091831
Gene Ontology: Biological Process	epithelial cell development	GO:0002064	0,0083	ENSG00000105976,ENSG00000082175,ENSG00000091831
Gene Ontology: Biological Process	intracellular receptor signaling pathway	GO:0030522	0,0157	ENSG00000082175,ENSG00000140009,ENSG00000091831
Gene Ontology: Biological Process	epithelial tube morphogenesis	GO:0060562	0,0321	ENSG00000105976,ENSG00000082175,ENSG00000091831
Gene Ontology: Biological Process	response to steroid hormone	GO:0048545	0,0330	ENSG00000082175,ENSG00000140009,ENSG00000091831
KEGG Pathway	Endocrine resistance	KEGG:01522	0,0007	ENSG00000141736,ENSG00000140009,ENSG00000091831
KEGG Pathway	Estrogen signaling pathway	KEGG:04915	0,0022	ENSG00000082175,ENSG00000140009,ENSG00000091831
KEGG Pathway	Proteoglycans in cancer	KEGG:05205	0,0074	ENSG00000105976,ENSG00000141736,ENSG000000918

				31
KEGG Pathway	Chemical carcinogenesis - receptor activation	KEGG:05207	0,0080	ENSG00000082175,ENSG00000140009,ENSG00000091831
Reactome Pathway	Nuclear Receptor transcription pathway	REAC:R-HSA-383280	0,0001	ENSG00000082175,ENSG00000140009,ENSG00000091831
Reactome Pathway	ESR-mediated signaling	REAC:R-HSA-8939211	0,0159	ENSG00000082175,ENSG00000140009,ENSG00000091831
Reactome Pathway	Signaling by Nuclear Receptors	REAC:R-HSA-9006931	0,0380	ENSG00000082175,ENSG00000140009,ENSG00000091831
WikiPathway	Mammary gland development pathway - Puberty (Stage 2 of 4)	WP:WP2814	0,0000	ENSG00000141736,ENSG00000082175,ENSG00000091831
WikiPathway	Nuclear receptors	WP:WP170	0,0001	ENSG00000082175,ENSG00000140009,ENSG00000091831
Gene Ontology: Molecular Function	nuclear estrogen receptor activity	GO:0030284	0,0000	ENSG00000140009,ENSG00000091831
Gene Ontology: Molecular Function	estrogen response element binding	GO:0034056	0,0001	ENSG00000140009,ENSG00000091831
Gene Ontology: Molecular Function	transcription coactivator binding	GO:0001223	0,0085	ENSG00000082175,ENSG00000091831
Gene Ontology: Molecular Function	transmembrane receptor protein tyrosine kinase activity	GO:0004714	0,0192	ENSG00000105976,ENSG00000141736
Gene Ontology: Molecular Function	transmembrane receptor protein kinase activity	GO:0019199	0,0327	ENSG00000105976,ENSG00000141736
Gene Ontology: Molecular Function	ATPase binding	GO:0051117	0,0359	ENSG00000082175,ENSG00000091831
Gene Ontology: Biological	mammary gland branching involved in pregnancy	GO:0060745	0,0008	ENSG00000082175,ENSG00

Process				000091831
Gene Ontology: Biological Process	branching involved in mammary gland duct morphogenesis	GO:0060444	0,0092	ENSG00000082175,ENSG00000091831
Gene Ontology: Biological Process	mammary gland duct morphogenesis	GO:0060603	0,0191	ENSG00000082175,ENSG00000091831
Gene Ontology: Biological Process	cellular response to estradiol stimulus	GO:0071392	0,0282	ENSG00000140009,ENSG00000091831
Gene Ontology: Biological Process	mammary gland morphogenesis	GO:0060443	0,0407	ENSG00000082175,ENSG00000091831
Gene Ontology: Biological Process	ovulation cycle process	GO:0022602	0,0460	ENSG00000082175,ENSG00000091831
KEGG Pathway	Prolactin signaling pathway	KEGG:04917	0,0345	ENSG00000140009,ENSG00000091831
KEGG Pathway	Central carbon metabolism in cancer	KEGG:05230	0,0345	ENSG00000105976,ENSG00000141736
KEGG Pathway	Adherens junction	KEGG:04520	0,0354	ENSG00000105976,ENSG00000141736
KEGG Pathway	Non-small cell lung cancer	KEGG:05223	0,0364	ENSG00000105976,ENSG00000141736
KEGG Pathway	EGFR tyrosine kinase inhibitor resistance	KEGG:01521	0,0439	ENSG00000105976,ENSG00000141736
Reactome Pathway	TFAP2 (AP-2) family regulates transcription of growth factors and their receptors	REAC:R-HSA-8866910	0,0036	ENSG00000141736,ENSG00000091831
Reactome Pathway	Sema4D in semaphorin signaling	REAC:R-HSA-400685	0,0093	ENSG00000105976,ENSG00000141736
Reactome Pathway	SUMOylation of intracellular receptors	REAC:R-HSA-4090294	0,0093	ENSG00000082175,ENSG00000091831
Reactome Pathway	Nuclear signaling by ERBB4	REAC:R-HSA-1251985	0,0157	ENSG00000082175,ENSG00000091831
Reactome Pathway	Transcriptional regulation by the AP-2 (TFAP2) family of transcription factors	REAC:R-HSA-8864260	0,0237	ENSG00000141736,ENSG00000091831
Reactome Pathway	Signaling by ERBB4	REAC:R-HSA-	0,0481	ENSG00000082175,ENSG00

		1236394		000091831
WikiPathway	Extracellular vesicle-mediated signaling in recipient cells	WP:WP2870	0,0112	ENSG00000105976,ENSG00000141736
WikiPathway	Ovarian infertility	WP:WP34	0,0128	ENSG00000082175,ENSG00000140009
WikiPathway	Oxysterols derived from cholesterol	WP:WP4545	0,0290	ENSG00000140009,ENSG00000091831
Gene Ontology: Molecular Function	hepatocyte growth factor receptor activity	GO:0005008	0,0500	ENSG00000105976

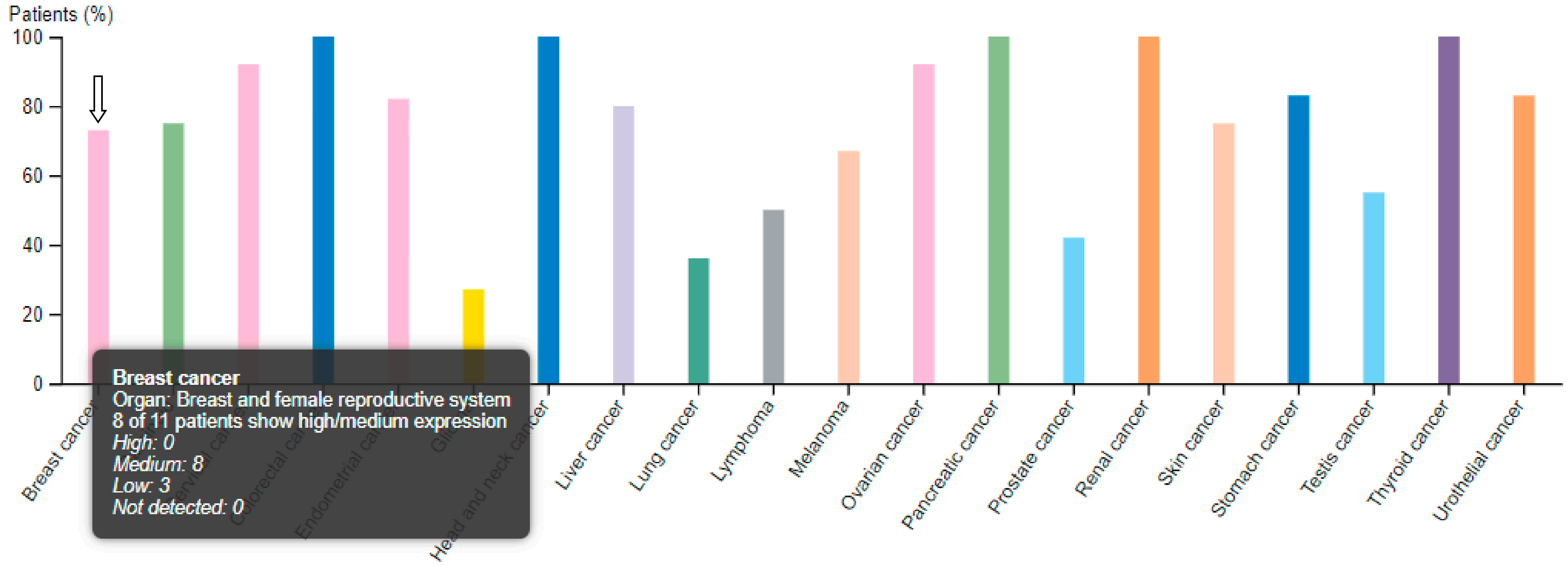

Figure S1. High expression of c-MET in Breast Cancer patients studied using Human Protein Atlas database.
